# Development of a machine learning-based acuity score prediction model for virtual care settings

**DOI:** 10.1186/s12911-023-02307-z

**Published:** 2023-10-03

**Authors:** Justin N. Hall, Ron Galaev, Marina Gavrilov, Shawn Mondoux

**Affiliations:** 1 Department of Emergency Services, C753, Sunnybrook Health Sciences Centre, Toronto, ON M4N 3M5 Canada; 2 Division of Emergency Medicine, Department of Medicine, Temerty Faculty of Medicine, University of Toronto, Toronto, ON Canada; 3 Institute of Health Policy, Management and Evaluation, University of Toronto, Toronto, ON Canada; 4 EmergConnect, Thornhill, ON Canada; 5 Department of Emergency Medicine, St. Joseph’s Healthcare Hamilton, Hamilton, ON Canada; 6 Division of Emergency Medicine, Department of Medicine, McMaster University, Hamilton, ON Canada

**Keywords:** Machine learning, Prediction model, Acuity scores, Remote triage, Virtual care

## Abstract

**Objective:**

Healthcare is increasingly digitized, yet remote and automated machine learning (ML) triage prediction systems for virtual urgent care use remain limited. The Canadian Triage and Acuity Scale (CTAS) is the gold standard triage tool for in-person care in Canada. The current work describes the development of a ML-based acuity score modelled after the CTAS system.

**Methods:**

The ML-based acuity score model was developed using 2,460,109 de-identified patient-level encounter records from three large healthcare organizations (Ontario, Canada). Data included presenting complaint, clinical modifiers, age, sex, and self-reported pain. 2,041,987 records were high acuity (CTAS 1–3) and 416,870 records were low acuity (CTAS 4–5). Five models were trained: decision tree, k-nearest neighbors, random forest, gradient boosting regressor, and neural net. The outcome variable of interest was the acuity score predicted by the ML system compared to the CTAS score assigned by the triage nurse.

**Results:**

Gradient boosting regressor demonstrated the greatest prediction accuracy. This final model was tuned toward up triaging to minimize patient risk if adopted into the clinical context. The algorithm predicted the same score in 47.4% of cases, and the same or more acute score in 95.0% of cases.

**Conclusions:**

The ML algorithm shows reasonable predictive accuracy and high predictive safety and was developed using the largest dataset of its kind to date. Future work will involve conducting a pilot study to validate and prospectively assess reliability of the ML algorithm to assign acuity scores remotely.

**Supplementary Information:**

The online version contains supplementary material available at 10.1186/s12911-023-02307-z.

## Introduction

Healthcare is becoming increasingly digitized, with mobile health and digital healthcare solutions becoming common. In Canada, 76% of Canadians believe that digital health has made healthcare more accessible and convenient, while more than 80% of Canadians are willing to use digital health services to view their medical records and access healthcare services [1]. The SARS-CoV-2 pandemic has accelerated patients’ willingness to access care through digital and virtual means [2]. While virtual care utilization has increased across the healthcare system, it remains underutilized in the emergency department (ED) [3, 4].

There are myriad potential contributors to the underutilization of virtual care in the ED such as acuity of patient presentations and absence of a triage system, debate on whether virtual care has a place in ED medicine, access to sufficient staffing support, and lack of referral pathways for patient investigations [5]. Simultaneously, as EDs experience increased patient volumes and overcrowding becomes the norm, patient safety and quality of care may be compromised [6]. Specifically, ED overcrowding may contribute to poor patient outcomes, worse patient experience, and increased lengths of stay [7–10]. Several solutions aimed at increasing ED efficiency including resource allocation and optimization technology, clinician activity and movement monitoring technology, and static wait time prediction systems, have been previously trialed with limited success [11–13].

Digital technologies that enhance the patient and provider experience by guiding patients to an appropriate care location based on their acuity has potential to increase virtual care utilization in the urgent care setting while simultaneously addressing some of the access and overcrowding challenges described above. Currently, there is a lack of remote and automated triage prediction systems and most virtual urgent care programs currently operate without a triage system [14, 15], despite triage being the first step of in-person emergency healthcare [16]. A digital, automated remote triage system may result in several improvements for both patients and care providers, namely improved patient comfort, care and satisfaction, and decreased provider workload [17].

In Canada, the Canadian Triage and Acuity Scale (CTAS) is a clinically validated tool [18, 19] used to assign triage acuity scores for patients presenting to EDs. The assigned score indicates the severity of the patient’s condition and a recommended timeframe for physician first assessment [20]. This system was digitized in 2015 to create eCTAS, an algorithmic real time electronic triage decision support tool for the physical ED environment [21]. Despite digitization, the system remains incompatible with virtual care in its current form. Specifically, eCTAS uses a nursing assessment designed to complement clinical care using stored protocols that improve the speed of triage. Furthermore, it requires the input of vital signs for each clinical encounter, which are generally unavailable in the virtual care setting. Moreover, the stored protocols do not account for any clinical risk caused by potentially dangerous complaint combinations, rather relying on the clinician to make these connections. As such, clinical risk assessments remain the standard of care, and the final score assignment is left to the discretion of the triage clinician [22]. Thus, there remains a need for a remote triage system that assesses clinical risk by heuristically analyzing key patient predictors such as presenting complaints, age, sex, and self-reported severity, and synthesizes this data into an accurate triage score.

Triage is a critical element of providing safe care in the virtual setting [23]. Patient-facing technologies that enhance the ability of patients to make informed decisions as to the most appropriate care location increase the safety of delivering virtual care compared to no triaging. Technology-based systems have demonstrated remote decision-making ability to triage patients to the appropriate level of care [24]. Machine learning (ML) models such as Deep Learning, Random Forest, and Naive Bayes are effective at predicting clinical outcomes through classification of triage urgency and discrimination between high and low case severity according to its clinical outcome [25]. Beyond Canada, ML has been used to assign triage scores in a clinical setting without clinician intervention with increased accuracy [26]. A multisite, retrospective, cross-sectional study used triage data to create a random forest model that predicted the need for critical care, an emergency procedure, and inpatient hospitalization while translating risk to triage level designations [26]. This study used the United States Emergency Severity Index (ESI) guidelines to train the ML model, demonstrating the potential use of machine learning in triage.

Virtual triage solutions have additionally been identified as a gap within the current virtual urgent care landscape in Canada and beyond [27, 28]. The objective of the current study is to describe the development of a ML-based acuity score prediction model that can be piloted in the Canadian context.

## Methods

### Study design and setting

This prediction model development study used retrospective patient-level encounter data for ED visits from three healthcare organizations in the Greater Toronto and Hamilton Area (GTHA) of Ontario, Canada: St. Joseph’s Healthcare Hamilton (SJHH; 70,000 annual visits), Sunnybrook Health Sciences Centre (SHSC; 64,000 annual visits), and William Osler Health System (WOHS; 200,000 annual visits). Standardized, de-identified, patient-level encounter data included presenting complaint(s), clinical modifiers, age, sex, self-reported pain, and nursing assigned CTAS triage scores. Vital sign metrics were not included as they are rarely available in the virtual setting. All data elements were extracted directly, after ethics approval and appropriate data sharing agreements, from the electronic health record tracking systems used at each site. The total number of patient encounters included was 2,460,109 (199,988 from January 2017 to December 2020 from SJHH, 125,543 from January 2018 to December 2020 from SHSC, and 2,134,578 from January 2011 to December 2020 from WOHS). 1252 visits were excluded due to a missing CTAS score (2,458,857 remaining records). 2,041,987 records were high acuity (CTAS 1–3) and 416,870 records were low acuity (CTAS 4–5). Demographics for the dataset are presented in Table 1. 75% of the dataset was used to derive the algorithm while 25% was reserved for accuracy testing. No personal health information (PHI) was extracted as part of the data collection. The study was approved and consent waiver granted by the Hamilton Integrated Research Ethics Board, the Sunnybrook Health Sciences Centre Research Ethics Board, and the William Osler Health System Research Ethics Board.

**Table 1:** Study Site Demographics and Canadian Triage and Acuity Scale (CTAS) Distributions

Demographics	SJHH	SHSC	WOHS	Total
Total Records, n	199,988	125,543	2,134,578	2,460,109
Men, n (%)	92,303 (46.2%)	56,234 (44.8%)	1,020,579 (47.8%)	1,169,116 (47.5%)
Women, n (%)	107,685 (53.8%)	69,309 (55.2%)	1,113,999 (52.2%)	1,290,993 (52.5%)
CTAS 1, n (%)	1514 (0.8%)	1084 (0.9%)	6209 (0.3%)	8807 (0.4%)
CTAS 2, n (%)	60,343 (30.2%)	30,347 (24.2%)	575,056 (26.9%)	665,746 (27.1%)
CTAS 3, n (%)	111,084 (55.5%)	72,704 (57.9%)	1,183,646 (55.5%)	1,367,434 (55.6%)
CTAS 4, n (%)	17,957 (9.0%)	19,923 (15.9%)	310,407 (14.5%)	348,287 (14.2%)
CTAS 5, n (%)	7846 (3.9%)	1485 (1.2%)	59,252 (2.8%)	68,583 (2.8%)
Missing CTAS, n (%)	1244 (0.6%)	0 (0%)	8 (0%)	1252 (0%)

Note: Due to rounding, totals may not sum to 100%

### Model derivation

The pattern-directed ML remote triage model was developed and trained using the retrospective, de-identified, patient-level encounter dataset. Since a CTAS score is assigned for each visit a patient makes to the ED, independent of any previous visits, each encounter record reflects a single cross-sectional point in time. CEDIS (Canadian Emergency Department Information System) [29] presenting complaints, relevant clinical modifiers, age, sex, and self-reported pain were used to predict acuity scores. The outcome variable of interest was the acuity score predicted by the ML system compared to the CTAS score assigned by the triage nurse. Five models were trained: decision tree, k-nearest neighbors, random forest, gradient boosting regressor, and neural net (using DataRobot’s AutoML feature in which a neural network is generated automatically and then optimized based on the specific dataset). Feature imputation was used during the training process to substitute missing self-reported pain score with average values by CEDIS complaint across the dataset (although this technique was not used as part of the final model).

### Model testing

Confusion matrices, precision, recall, and F-1 scores were used to assess the accuracy of predictions at each stage of testing using the reserved dataset. The two ML models with the greatest predictive accuracy were further refined by adjusting hyperparameters (n_estimators, learning_rate, max_depth, min_samples_split, n_iter_no_change). Grid search was used to determine the best hyperparameter for each model. After these refinements, the models were initialized and trained from scratch and then re-tested using the reserved dataset. The single ML model demonstrating the greatest predictive accuracy was selected.

**Fig. 1:**
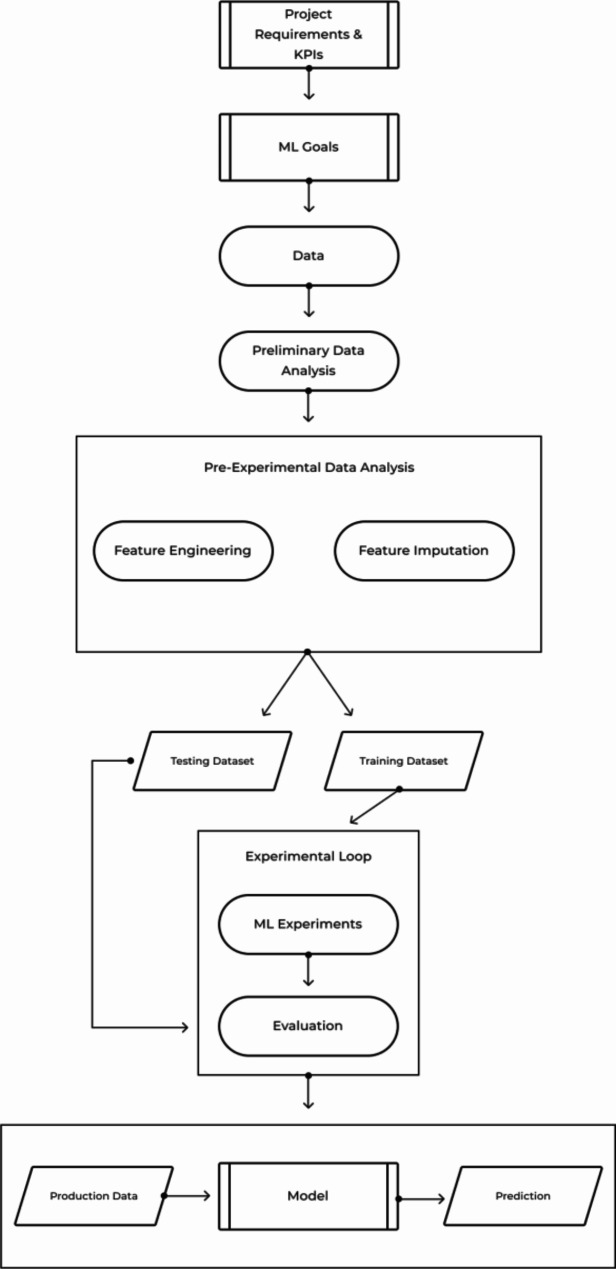
Workflow diagram showing machine learning algorithm development and testing process Where: KPI = key performance indicators, ML = machine learning

A confusion matrix is used to describe the performance of a classification model based on a set of test data for which the true values are known. The table layout allows for the visualization of performance of the model, with each row representing a known value in the testing dataset, and each column representing a predicted value. Each of the plots were reviewed continuously to determine the best learning parameters for the model and to avoid possible overfitting during development. Precision (or positive predictive value) is the proportion of predicted CTAS scores that are correct and is calculated by taking the proportion of true positive values (i.e., true CTAS 3) to the sum of the true positive and false positive values (i.e., true CTAS 3 and falsely predicted CTAS 3). Recall (or sensitivity) is the total proportion of correct predictions and is calculated as the proportion of true positive values (i.e., true CTAS 3) to the sum of true positive and false negative values (i.e., true CTAS 3 and incorrectly predicted other CTAS). Recall is an important parameter as false negative triage scores have the possibility of significant downstream patient harm. The F1 score is a weighted average of precision and recall and considers both false positives and false negatives (ranges from 0 to 1 with a higher score being better). To decrease potential safety risk of missing a high acuity presentation, the distribution of CTAS presenting complaints within the dataset was reviewed manually and all complaints with a 1% or higher distribution of CTAS 1 scores were up triaged such that the final model predicts a CTAS 1 score for all these complaints. A workflow diagram showing model development is displayed in Fig. 1.

## Results

**Fig. 2:**
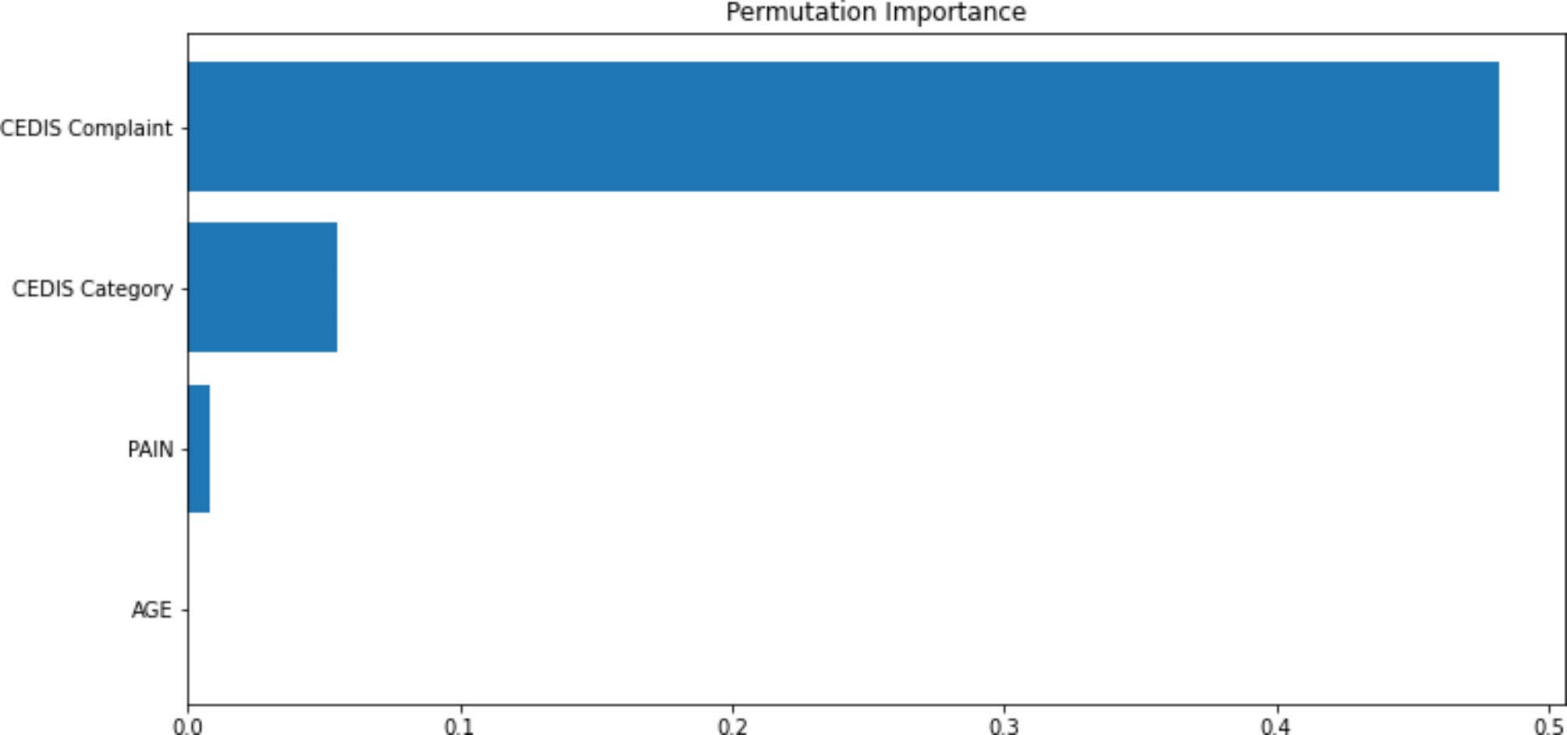
Permutation importance plot showing features with highest importance in the final ML model

**Fig. 3:**
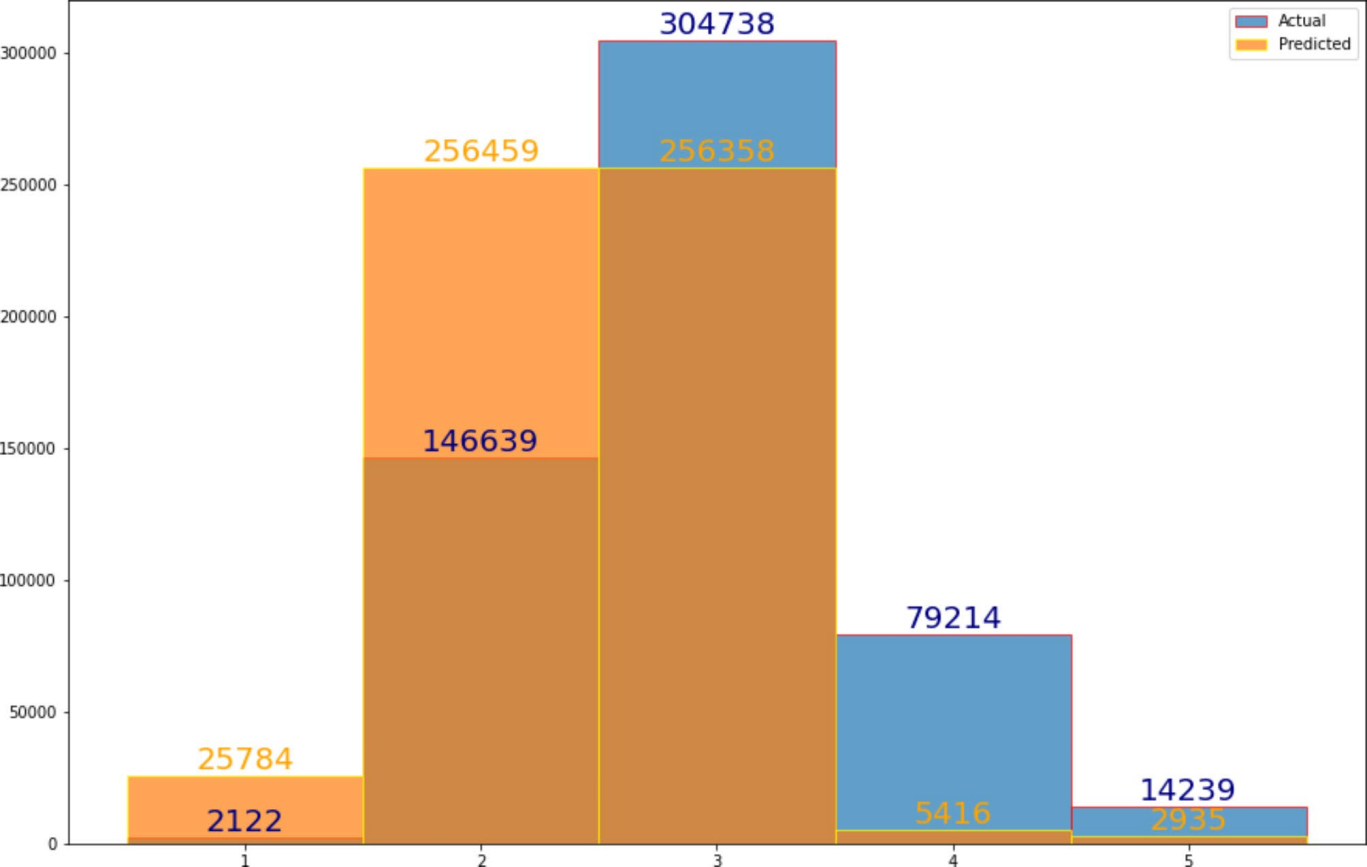
Distribution of actual CTAS (blue) and predicted acuity scores with the up triaging of CTAS 1 complaints using the 1% cutoff (orange) from the test set

**Fig. 4:**
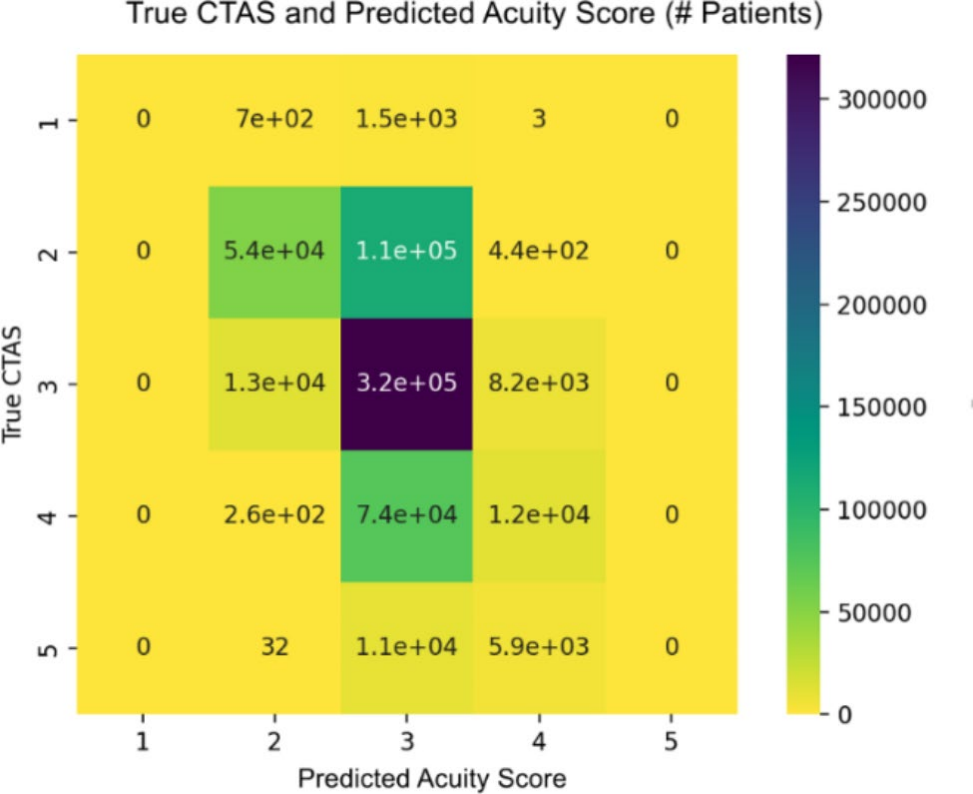
Final model confusion matrix with rows representing true CTAS scores and columns representing predicted acuity scores

The five initial ML models (decision tree, k-nearest neighbors, random forest, gradient boosting regressor, and neural net) were tested with the random forest and gradient boosting regressor demonstrating the greatest predictive accuracy. After adjusting hyperparameters, the gradient boosting regressor was shown to be the model that provided the most accurate predictions. The final ML model includes the following data elements: CEDIS complaint, CEDIS category (umbrella grouping of CEDIS complaints such as cardiovascular or neurologic), pain level, and age (Fig. 2). Figure 3 displays the distribution of actual CTAS scores as compared to the predicted acuity scores using the 1% up triaging cut-off for CTAS 1 scores for the test set. Figure 4 shows the confusion matrix illustrating the final model was best at predicting CTAS scores of 3 and worst at predicting CTAS scores of 1 and 5.

Table 2 displays the precision, recall, and F1 scores for the final model. The overall average represents the calculated average with equal weighting to each acuity score category whereas the weighted average is the calculated average based on the proportion of each acuity score category within the dataset. Based on the F1-score, the model performs best for patients with a CTAS score of 3, and worst for those with CTAS scores of 1 or 5.

**Table 2:** Precision and Accuracy of Final Model Using Test Set

	Precision	Recall	F1 Score	Total Patients
CTAS 1	0.00	0.00	0.00	2242
CTAS 2	0.79	0.32	0.46	167,815
CTAS 3	0.62	0.94	0.74	343,559
CTAS 4	0.46	0.14	0.21	86,933
CTAS 5	0.00	0.00	0.00	17,101
Accuracy			0.63	617,650
Overall Average	0.37	0.28	0.28	617,650
Weighted Average	0.62	0.63	0.57	617,650

The final model shows an overall accuracy for one-to-one predictions of 47.4%. One-to-one accuracy refers to the testing dataset containing the exact triage score predicted for the specific test case. Due to the subjectivity of triage scores, inconsistencies were identified within the retrospective dataset. Therefore, the model was tuned to predict more acute triage scores in ambiguous situations, excluding CTAS 1 as this is the most acute triage score possible, leading to an overall accuracy of equal to or more acute triage scores of 95.0%. Tables 3, 4 and 5 display summaries of the final model prediction statistics (overall, by CTAS score, and by CEDIS presenting complaint (top 10 most common presenting complaints)). Table 6 displays the CEDIS presenting complaints that were up triaged to CTAS 1 in the final ML model. Appendix 1 shows the final model prediction statistics for all CEDIS presenting complaints.

**Table 3:** Final Post-Processing Model Overall Prediction Statistics

Parameter	Total %	Non-Equal Triage Subset %
Equal Triage	47.4	N/A
**Non-Equal Triage**	52.6	100
***Up Triaged***	**47.6**	**90.5**
One-Step	37.9	72.1
Two-Step	8.1	15.4
Three-Step	1.6	3.0
***Down Triaged***	**5.0**	**9.5**
One-Step	4.7	8.9
Two-Step	0.3	0.6
**Equal and/or Up Triaged**	**95.0**	** N/A**

**Table 4:** Final Post-Processing Model Prediction Statistics by CTAS Score

Parameter	CTAS 1%	CTAS 2%	CTAS 3%	CTAS 4%	CTAS 5%
**Equal Triage**	7.9	75.8	48.5	1.1	0.6
**Up Triaged**	N/A	8.5	50.7	98.4	99.4
One-Step	N/A	8.5	42.4	79.6	18.5
Two-Step	N/A	N/A	8.4	10.4	74.8
Three-Step	N/A	N/A	N/A	8.4	5.9
**Down Triaged**	92.1	15.7	0.7	0.5	N/A
One-Step	88.0	15.7	0.5	0.5	N/A
Two-Step	4.1	0	0.2	N/A	N/A

Note: Due to rounding, totals may not sum to 100%

**Table 5:** Final Post-Processing Model Prediction Statistics by CEDIS Presenting Complaint for Top 10 Most Common CEDIS Complaints

CEDIS Presenting Complaint	Equal Triage %	Up Triage %				Down Triage %			Total %
		Total	One Step	Two Step	Three Step	Total	One Step	Two Step	
Abdominal Pain	52.8	41.9	38.0	3.7	0.2	5.3	5.3	0	100
Chest Pain – Cardiac Features	52.2	42.8	38.9	3.7	0.2	5.0	5.0	0	100
Upper Extremity Injury	52.7	42.0	38.0	3.8	0.2	5.3	5.3	0	100
Lower Extremity Injury	52.8	42.1	38.3	3.7	0.1	5.1	5.1	0	100
Fever	52.7	42.1	38.0	4.0	0.1	5.1	5.1	0	99.9
Lower Extremity Pain	53.0	41.7	38.0	3.5	0.2	5.2	5.2	0	99.9
Shortness of Breath	2.4	97.2	27.4	55.7	14.1	0	0	0	99.6
Back Pain	52.6	42.0	38.4	3.5	0.1	5.4	5.4	0	100
Headache	53.1	41.8	37.8	3.8	0.2	4.9	4.8	0.1	99.8
Laceration/Puncture	51.5	41.4	37.6	3.7	0.1	6.7	5.3	1.4	99.6

Note: Due to rounding, totals may not sum to 100%

**Table 6:** CEDIS Presenting Complaints Up Triaged to CTAS 1 Showing Proportion of CTAS 1 Scores within Dataset

CEDIS Presenting Complaint	CTAS 1 Proportion (%)
Stridor	50.0%
Cardiac Arrest (non-traumatic)	36.4%
Cardiac Arrest (traumatic)	28.6%
Cool Pulseless Limb	16.7%
Altered Level of Consciousness	3.5%
Isolated Chest Trauma – Penetrating	3.2%
Multisystem Trauma – Penetrating	3.1%
Violent/Homicidal Behaviour	2.6%
Amputation	2.0%
Hypoglycemia	1.3%
Bizarre Behaviour	1.2%

## Discussion

This study advances our knowledge around the potential use of remote and virtual triage for Canadian emergency departments in three important ways. First, our ML model system can predict one-to-one triage scores with a moderate degree of accuracy based on set patient predictors. Recently published findings about emergency department-led virtual urgent care from Ontario Canada has demonstrated that most (> 92%) patients using these services are CTAS 3–5 or moderate to lower acuity patients [15]. Thus, based on the typical patients that use these virtual care services, the safety profile of the current algorithm is high as it is most accurate for moderate acuity scores, and it over predicts acuity in most patients where the prediction is not equal to the actual acuity score. This builds on previous work which has demonstrated the ability of ML algorithms to accurately discriminate between high and low presentation severity and expected clinical outcomes [25, 26, 30, 31]. Of particular importance is that the current work is the first study to use Canadian data including CTAS and is also the largest study of its kind to date (each of the above studies developed ML models with fewer than 200,000 patient records whereas the ML model from the current work is based on more than 2.4 million patient records).

Second, the subjective nature of triage score assignment was observed in the retrospective dataset where patients with identical predictors were assigned different triage scores both within the same site and between different sites. This phenomenon has been well-described in the published literature with several studies demonstrating triage score variability through individual and departmental audit and feedback, variations in intensity and duration of training programs, and simulated case scenarios with missing modifiers, inappropriate manual down/up triage override rates, and variable clinical triage experience contributing to the observed variability [20, 21, 32–38]. A CTAS triage meta-analysis showed a 42.82% mis-triage rate across studies (25.52% up triage and 17.30% down triage) with most up triages being clinically plausible and down triages posing a greater risk to patient safety [20]. Additionally, Kovacs and Campbell [39] showed a 3% increased probability that a triage nurse assigned higher triage acuity scores compared to triage paramedics in non-crowded ED conditions and a 10% higher probability of assigning a more acute triage score in crowded ED conditions. Moreover, recent work has shown that eCTAS systemically reclassifies patients from higher to lower acuity scores [40].

To address this limitation, the current algorithm builds predictions with greater accuracy through model tuning and requiring the model to assign more acute triage scores in ambiguous cases. This was further refined by using a gradient boosting algorithm that predicts incremental triage scores. These refinements serve to minimize the potential patient safety risk of under-triaging (47.6% up triage rate and only a 5.0% down triage rate). Thus, the current ML algorithm has the potential to overcome some of this triage score variability and may contribute to decreased provider workload and improved patient safety, particularly during periods of ED overcrowding.

Third, by using artificial intelligence to analyze the current study’s large retrospective dataset, the current work distinguishes which features of a patient profile most influence triage score assignment and incorporates these features into its ML prediction. While feature importance has not been published previously related to predicting triage scores, its use has been demonstrated related to predicting clinical outcomes such as need for hospitalization or critical care setting for patients presenting to the ED [30]. By identifying which patient profile features are most important to determining an accurate assessment of triage acuity, the current work provides direction for the development of future patient-facing applications that can incorporate these elements into their design, thereby reducing risk associated with virtual care options that largely only use self-triage currently. It should be noted that this weighting includes only those features that are already used by eCTAS and not alternative elements within the patient chart, as discussed below.

There are, however, a few important limitations of the current work. The first is the lack of access to subjective data related to the triage experience and nursing expertise. For example, triage scores may be influenced by patient features such as physical appearance, previous health history that is not captured within the past medical history section, recent ED visits or hospitalizations, regularity of patient visits, the nurse’s gestalt, among others [20, 34, 36]. This narrative data is not always captured within electronic health records as part of the triage process beyond the acuity score assignment and was not available for analysis and training in this study. Inclusion of this narrative data based on the nurse’s expertise may enhance the predictive ability of the ML system in the future as it has recently been shown to help predict patient disposition based on emergency triage notes [41]. Natural language processing (NLP) of the triage complaint and integration within the algorithm should be explored in future work.

Additionally, the final model before post-processing performs the best for patients with CTAS scores of 3 and worst for those with CTAS scores of 1 and 5. This is consistent with other literature that has shown ML algorithms outperforming traditional triage methods for patients with moderate scores [26, 42]. This may be partially attributed to the class imbalance in the dataset as most patients, 55.6%, in our evaluation dataset have a CTAS score of 3. This predominance of CTAS 3 is consistent with previously published CTAS reviews [35, 37, 38]. The inability of the algorithm to accurately assign acuity scores to CTAS 5 patients poses minimal patient safety risk; however, assigning lower acuity scores for CTAS 1 patients may present a patient safety risk. We sought to address this limitation by manually up triaging all CEDIS presenting complaints that had 1% or more CTAS 1 scores within the derivation dataset. This resulted in 11 CEDIS complaints being up triaged to CTAS 1 as shown in Table 6. The final model was also tuned to predict more acute triage scores in ambiguous situations, excluding CTAS 1 as this is the most acute triage score possible, thus post-processing, the model performs best for patients with a CTAS score of 2 and worst with a CTAS score of 5 (2 -> 3 -> 1 -> 4 -> 5). Consideration will need to be made to determine how best to pilot this ML algorithm to ensure patient safety, such as directing acuity scores of 1 or 2 to emergency medical services emergently for medical attention. It is also important to note that patients who are classified as CTAS 1 are unlikely to seek out virtual care, or pass the screening questions to recommend this type of care, further decreasing the potential risk of this tool. Future research may place more emphasis on collecting data from the other, less commonly assigned groups, or exploring the effect of up-sampling on model performance. Rather than predicting an exact acuity score to parallel the CTAS score, a model which provides clinical direction, such as in-person ED, virtual urgent care, or family doctor/walk-in clinic may be beneficial for patients.

Further model improvements may be possible. ML model accuracy is generally computed based on the number of correct and incorrect predictions. The current model has gone a step further by evaluating outcomes in a non-binary way to reflect ‘how incorrect’ a prediction is. Variable penalties are applied based on the degree of inaccuracy of the prediction, in which the more incorrect a prediction is, the greater the penalty. This was put in place due to the ambiguity and subjectivity found in the dataset. In practice, this means that triage scores predicted are not only more likely to be accurate but also more acute rather than less acute. This is analogous to how in a clinical setting, in ambiguous cases, triage nurses assign more acute scores rather than less acute triage scores to safeguard patient safety [20]. A possible future direction is to use a quantile regression approach to build confidence prediction intervals rather than a single acuity score. Another is to test this relative to a simulated dataset with 100% triage accuracy as defined by eCTAS.

Overall, the current ML algorithm provides reasonable predictive accuracy and high predictive safety specific to the patient population that generally accesses emergency department-led virtual urgent care services. This affords great potential for implementation as part of a broader machine learning driven patient facing system for remotely assigning triage scores in an area where there is no current active triage system. The ability of the system to remotely assign accurate acuity scores offers promise in helping triage patients to the best care location and decreases potential risk associated with virtual urgent care programs.

### Electronic supplementary material

Below is the link to the electronic supplementary material.

Supplementary Material 1

## Data Availability

The data are only available to the collaborating scientists from the respective participating centres. The data may be available upon request for some of the participating centres but not all due to relevant organization-specific data protection policies.
